# Bounding systems: A qualitative study exploring healthcare coordination between the emergency youth shelter system and health system in Toronto, Canada

**DOI:** 10.1371/journal.pone.0303655

**Published:** 2024-06-21

**Authors:** Alzahra Hudani, Kaitlin Schwan, Ronald Labonté, Sanni Yaya

**Affiliations:** 1 Interdisciplinary School of Health Sciences, University of Ottawa, Ottawa, Ontario, Canada; 2 Women’s National Housing & Homelessness Network, Toronto, Ontario, Canada; 3 School of Epidemiology and Public Health, University of Ottawa, Ottawa, Ontario, Canada; 4 School of International Development and Global Studies, University of Ottawa, Ottawa, Ontario, Canada; 5 The George Institute for Global Health, Imperial College London, London, United Kingdom; Sinai Health System, CANADA

## Abstract

**Background:**

Several youth staying at emergency youth shelters (EYSs) in Toronto experience poorly coordinated care for their health needs, as both the EYS and health systems operate largely in silos when coordinating care for this population. Understanding how each system is structurally and functionally bound in their healthcare coordination roles for youth experiencing homelessness (YEH) is a preliminary step to identify how healthcare coordination can be strengthened using a system thinking lens, particularly through the framework for transformative system change.

**Methods:**

Forty-six documents, and twenty-four semi-structured interviews were analyzed to explore how the EYS and health systems are bound in their healthcare coordination roles. We continuously compared data collected from documents and interviews using constant comparative analysis to build a comprehensive understanding of each system’s layers, and the niches (i.e., programs and activities), organizations and actors within these layers that contribute to the provision and coordination of healthcare for YEH, within and between these two systems.

**Results:**

The EYS and health systems are governed by different ministries, have separate mandates, and therefore have distinct layers, niches, and organizations respective to coordinating healthcare for YEH. While neither system takes sole responsibility for this task, several government, research, and community-based efforts exist to strengthen healthcare coordination for this population, with some overlap between systems. Several organizations and actors within each system are collaborating to develop relevant frameworks, policies, and programs to strengthen healthcare coordination for YEH. Findings indicate that EYS staff play a more active role in coordinating care for YEH than health system staff.

**Conclusion:**

A vast network of organizations and actors within each system layer, work both in silos and collaboratively to coordinate health services for YEH. Efforts are being made to bridge the gap between systems to improve healthcare coordination, and thereby youths’ health outcomes.

## Background

Approximately 6, 000 to 7, 000 youth between the ages of 13–24 experience homelessness in Canada on any given night [1]. The adversity of suffering from homelessness exposes youth to a multitude of physical, emotional, and mental health concerns. First, youth experiencing homelessness (YEH) are exposed to inadequate nutrition and sleep, high levels of stress, poor hygiene, increased risk of injury, greater exposure to sexually transmitted infections due to increased sexual activity with more partners, and greater exposure to a range of other infectious diseases; all of which lead to poor overall health [2–4]. A few North American studies have shown that YEH are 6 to 12 times more likely to suffer from HIV and are more likely to contract Chlamydia than stably housed youth [5, 6]. Other studies have shown that respiratory disease is more prevalent among YEH, which may be associated with residence in crowded areas including emergency youth shelters (EYSs) [7, 8]. Second, most YEH face a myriad of mental health concerns such as depression, anxiety, post-traumatic stress disorder, and paranoia [9]. Poor mental health is one of the most prominent and complex concerns among Canadian YEH, as the experience of being homeless is associated with deteriorating mental health, and mental health and addictions often lead to being homeless [10]. From the sample of youth (N = 1,375) who participated in the second and most recent national youth homelessness survey (2019), 74% fell in the ‘high’ symptom/distress category on the GAIN short screener mental health survey, 35% reported at least one suicide attempt, and 33% reported at least one overdose requiring hospitalization [11]. These findings align with findings from the first national youth homelessness survey (N = 1,103) (2016) [1, 10], and other studies that show suicide and drug overdose to be leading causes of mortality among YEH in Canada. For example, a large cohort study in Montreal (N = 1,013) found that the mortality rate of YEH was 11 times higher than youth who were stably housed, with suicide and drug overdose being the two leading causes of death [4]. In addition to suffering from poorer health outcomes, YEH experience significantly poorer access to health services than stably housed youth [12, 13].

Findings from the first national youth homelessness survey indicate that 55.5% of YEH live transiently in multiple, precarious locations with about a third residing at EYSs [1]. YEH may access health services at EYSs depending on each shelter’s respective infrastructure and capacity to provide health services for their individual health needs. In addition to accessing health services integrated within EYSs, youth may choose to access healthcare externally through youth drop-in centres, their family doctor, hospital emergency departments, and/or walk-in clinics [14]. However, when facing crises (e.g., overdosing) for which Toronto-based shelter staff are not equipped to provide care, youth are often sent to receive this care externally; usually to the nearest hospital emergency department [15]. Evidence from a recent study has shown that YEH often get trapped in a vicious shelter-hospital loop, which commonly occurs when they are sent from an EYS to receive emergency care at hospitals. These youth are often prematurely discharged back into the EYS system, only to later re-enter the health system for health concerns or crises for which they were previously admitted. This critical disconnect results from insufficient engagement between systems, and highlights the need for strengthened healthcare coordination between the EYS and health systems to improve YEH’s pathways to care and ultimately their health outcomes [15].

Healthcare coordination is defined by the Agency for Healthcare Research and Quality as the “*The deliberate organization of patient care activities between two or more participants (including the patient) involved in a patient’s care to facilitate the appropriate delivery of healthcare services*.*”* Despite the critical need for strengthened coordination within and between the EYS and health systems, there are many barriers that prevent adequate, inter-organizational engagement [16]. Some of these barriers include but are not limited to neither system being solely responsible for helping youth navigate continuous healthcare beyond their stay or visit within either sector, poor communication between system staff due to prohibitive privacy and information-sharing policies, even within circles of care; differences in internal organizational healthcare coordination processes across each sector, and the fragmented evolution of these two systems over time [15, 17, 18]. These examples are a few of many interconnected factors that make it challenging for YEH to navigate healthcare without support [18–20].

The unfortunate experience of navigating disconnected government-funded public systems (e.g., education, social services, health services, and shelter, support and housing services), have left many YEH with gaps in their care, and thereby a loss of trust in these systems [18, 21]. Additionally, several gaps and barriers exist in how these complex and siloed systems are governed and operate, exacerbating the challenge of sustainably coordinating care between systems [22, 23]. A few of these barriers include staffs’ limited knowledge of available resources, services, and practices within and between systems; and limited and inadequate access to post-discharge health and social services for their youth clients. For instance, a recent study found that shelter staff perceived hospital staff to have a limited understanding of the different specializations between shelters, with some being more accessible to them than others. This sometimes led to hospital staff inappropriately discharging their youth patients to shelters that were poorly suited for their healthcare needs. Similarly, shelter staff reported having a limited understanding of their clients’ healthcare needs and the types of issues acute care is most appropriately able to address [22]. Overall, poorly coordinated, or uncoordinated healthcare in the homeless youth serving sector can lead to youths’ entrapment in the shelter-hospital loop, making it increasingly difficult for YEH to improve their health outcomes and escape the vicious cycle of homelessness [15].

As a result, there is an urgent need to strengthen healthcare coordination between the EYS and health systems in Toronto [1]. Healthcare coordination involves supporting youth with accessing appropriate healthcare, synchronizing the delivery of their care across multiple providers between systems, sharing a care plan with youth and providers who fall within their circle of care, and coordinating required post-care through follow-up. Successful healthcare coordination within and between systems requires easy access to a range of healthcare services and providers, adequate communication between providers, and effective care plan transitions between providers [24]. To our knowledge, there are no studies that have explored how actors or organizations within the EYS and health systems individually or collectively engage to coordinate care for YEH in Toronto.

In this study, we aim to elucidate the various levels, niches (i.e., programs and activities), organizations, and actors that lie within each system with respect to their role in coordinating health services for YEH, particularly based on the 4-step framework for transformative systems change which is grounded in systems thinking and organizational change. “Bounding the system” is the first step of the 4-step framework developed by Foster-Fishman and colleagues, which aims to clarify how system(s) are conceptualized against their external environment. Steps 2–4 of the framework focus on understanding the fundamental parts of each system (norms–resources–regulations -operations) as potential root causes for poorly coordinated healthcare between the EYS and health systems, assessing system interactions through causal links and feedback loops, and identifying levers for change within each system, respectively [25].

Based on the framework for transformative systems change, establishing system boundaries is the first and possibly most pivotal and defining step to appropriately devise systems change interventions, in this case to strengthen healthcare coordination within and between the EYS and health systems for YEH. This is largely because boundary lines are known to have explicit values associated with them [26]. For instance, by clarifying who and what is enclosed within and outside boundary lines, explicit statements can be made about the perspectives, roles, and functions that are critical for coordinating care within and between each system [25]. Therefore, to understand the relationships, interactions, and interdependencies within and between parts of each system, which were investigated subsequently through steps 2–4 of the framework, it is important to first understand how each system is structurally and functionally bound in their healthcare coordination roles.

In bounding the EYS and health systems, we first identify system levels by outlining relevant layers within each of these systems that play a role in healthcare coordination for YEH in Toronto. We then highlight the niches, local organizations, and key actors that lie within each identified layer to develop a deeper understanding of how these systems are bound and who and what they entail [25]. Therefore, the overarching objective of this article is to **define the boundaries of the EYS system and health system, particularly in how they coordinate healthcare for YEH**. The research questions explored in response to this objective are two-fold: 1) How are the EYS system and health systems structurally and functionally bound in their roles coordinating healthcare for YEH in Toronto**?** and 2) How are components within the boundaries of each of these systems interconnected in their efforts to coordinate care for YEH?

## Methods

### Study setting & design

This qualitative analysis is part of a larger case study exploring how the EYS and health systems engage to coordinate care for the health needs of YEH in the inner-city and inner-suburban regions of Toronto; the capital city of the province of Ontario and most populous city in Canada with a population exceeding 6.4 million people [27].

We critically analyzed documents and key informant interview transcripts to identify the elements (layers–niches–organizations–actors) that structurally and functionally bind the EYS and health systems in how they individually and collectively coordinate healthcare for YEH in Toronto. While key informant interviews were initially being conducted as part of the larger case study, we realized that this data contained rich information about how each of system is bound, filling gaps in the document analysis. Many key informants spoke about their role in providing and coordinating healthcare for youth within either or both systems, including their relationships with other organizations and care providers. The study was then adapted to include both documents and key informant interview data to respond to the research questions. The document analysis and interviews have informed the other throughout the research process, as guided by the emergent properties of constant comparative analysis. Constant comparative analysis is a qualitative analytic method through which data is coded, categorized, and constantly revisited until no new themes or categories emerge [28].

### Data collection

#### Document analysis

The principle investigator (AH) extracted and analyzed 46 documents that helped define how the EYS and health systems are bound in their healthcare coordination roles for YEH using the 4-step READ approach: 1) Ready your materials, 2) Extract data, 3) Analyze data, and 4) Distil your findings [29]. Documents included organizational websites, reports, frameworks, brochures, program evaluations, policy briefs and legislation, and scholarly articles. Documents were purposively selected and sequentially reviewed followed by snowball sampling to select other relevant documents (see search strategy outlined in Table 1). All documents were filed into NVivo 12.0 software for subsequent analysis. Upon review, 4 documents with irrelevant or duplicate information were removed, resulting in 46 out of 50 documents being included in the analysis. Tables detailing which documents were considered for inclusion based on the search strategy, and which were excluded can be found in the supplementary file (S1 Table in S1 File) attached.

**Table 1:** Search strategy used for document analysis.

	Documents Source	Description
1	Organizational Websites	• Explored health-based programming for YEH across organizations within each system including EYSs, community health centers, hospitals, etc. through their respective organizational websites. The 10 EYS websites in the inner-city and inner suburban regions of Toronto were searched first, as were relevant health-based organizations that were involved or stated as shelter partners in providing health services for youth. • Reviewed reports from the websites, most of which outlined relevant programs, activities, and actors within and across each system. • Websites of relevant health-based organizations mentioned by key informants during in-depth interviews were also reviewed for inclusion in the document analysis.
2	Government Websites	• Scanned the City of Toronto’s webpage for housing and homelessness research and reports; as well as relevant meeting agendas, policies, and reports through their search bar [30]. Search terms included: EYSs, the Toronto Alliance to End Homelessness, Toronto Shelter Network and Youth Shelter Interagency Network. • Strategically searched for documents through the Province of Ontario website, using the following filter (Ministry: health, News type: all, Topic: health and wellness; home and community) [31].
3	Scholarly Literature	• Extracted relevant peer-reviewed articles from the MEDLINE database using a combination of specific search terms. • Search terms included: 1) Emergency Youth Shelter AND Toronto; and 2) (((health system) OR (hospital) OR (healthcare) OR (clinic) OR (community health)) AND ((Ontario) OR (Toronto)) AND ((youth) OR (adolescents) OR (young people)) AND (coordination)). • A filter was applied to include articles published between 2019- Present to ensure recent system-level reforms such as the establishment of Ontario Health were being captured. • A total of 8 out of 224 articles were selected for full review after carefully reviewing article titles for relevance. Four out of the 8 articles were then selected for inclusion and further analysis.
4	Documents from interviews	• Finally, private documents shared by key informants during interviews were included in the analysis. These documents included organizational protocols, policies, and/or frameworks relevant to coordinating healthcare for YEH.

### Key informant interviews

The principal investigator, AH, facilitated twenty-four semi-structured interviews with key informants over Microsoft Teams. Interviews took place between May 27^th^, 2021, and March 29^th^, 2022, and each ranged between 45 minutes to 1-hour in length. Interview guides were informed by Foster-Fishman and colleagues’ framework for transformative systems change and consisted of two separate guides developed for youth and non-youth participants. Interview questions were open-ended and focused on identifying and assessing healthcare coordination processes within and between the EYS and health systems in Toronto, and fundamental system parts (i.e., identifying system norms, resources, regulations, and operations) that influence these processes. For instance, non-youth interview participants (i.e., high-level executives and frontline staff) were asked about healthcare programs offered at their respective organizations, and the formal and informal relationships that exist in their healthcare coordination processes. Youth participants were asked about their process of seeking care for minor or major health concerns; the health professionals, programs, and organizations they interacted with; their experience with receiving care; any follow-up support received or not received post health care visit, etc. All interviews were recorded with participants’ consent and transcribed using Otter.ai software.

Participant recruitment was initiated through purposive sampling, where AH first contacted high-level executives and clinicians who worked within the EYS and health systems, respectively. She then used snowball sampling to recruit other interview participants including lower-level executives such as case managers, and various frontline staff with clinical (e.g., nurse, social worker, etc.) and non-clinical (e.g., program coordinators, outreach counsellors, etc.) backgrounds. Youth recruitment was supported by two frontline staff who worked at EYSs and one who worked in the community health sector. These staff supported recruitment by posting flyers at their respective organizations, and/or personally sharing flyers and information sheets with clients who met inclusion criteria. Youth were interviewed if they: 1) ranged between 16 and 24 years of age, 2) were currently or previously staying at a Toronto-based EYS, and 3) had experience navigating healthcare through health services integrated at an EYS; healthcare at organizations, agencies, or institutions within the health system; and/or organizations or healthcare providers that overlap between both systems. Table 2 displays a breakdown of interview participants who work within each system, and those whose work and/or experience overlaps between systems.

**Table 2:** Key informants interviewed for study.

Key Informant	EYS system	Overlap between systems	Health system
Executives	6	0	1
Frontline staff with clinical expertise	1	3	3
Other frontline staff	1	0	3
YEH	0	6	0

### Data analysis

Data from documents and interview transcripts were concurrently analyzed using two layers of coding: open coding and focused coding [32, 33]. During open coding, AH inductively coded documents and interview transcripts for layers, niches, organization, and/or actors within the EYS and health systems. These initial codes were then organized into more defined categories based on systems layers that had emerged. While conducting focused coding, AH recognized a significant overlap between systems, through which a third category of systems emerged. Each level of analysis required a reworking of the data to appropriately organize codes into the predetermined categories falling within the first component of Foster-Fishman and colleagues’ framework for transformative systems change: bounding systems. Additionally, AH simultaneously documented descriptive summaries and emergent patterns derived from the data through memos, which helped generate relationships between system elements. Data from documents and key informant interviews were constantly compared to comprehensively grasp how each system is bound and connected in their healthcare coordination roles for YEH in Toronto.

### Trustworthiness

Rigor in the qualitative research process is critical to produce trustworthy findings. To enhance the credibility and confirmability of this research, we used data source triangulation and memo-writing to support the analysis of incoming data [34]. First, in analyzing a range of scholarly and grey literature, and interview transcripts from five distinct groups of stakeholders form various levels of both systems, we were able to capture a wide range of system elements to comprehensively understand and define the boundaries of the EYS and health systems in their individual and collective healthcare coordination roles. Further, analytic memos were consistently and reflexively documented to compare and draw connections between data, while being mindful of any researcher biases, and/or thoughts and feelings that presented themselves during key informant interviews. Documents, interview transcripts, and analytic notes were revisited several times to confirm accuracy of study findings.

### Ethics

Research ethics approval was granted by the University of Ottawa Health Sciences and Sciences Research Ethics Board (See S2 File). Document analysis and semi-structured interviews were performed in accordance with the Declaration of Helsinki. All key informants voluntarily agreed to participate in interviews and provided verbal or written informed consent prior to their interview. Verbal consent was recorded using Otter.ai software prior to commencing interviews, and written consent was received through consent forms which were sent beforehand by email. AH ensured that all youth participants had access to a safe and private space from which to interview and that they were compensated $30 for their time and participation. All consent forms, interview recordings and transcripts are technically safeguarded on AH’s password protected computer and will be retained for 5 years as per the protocol approved by the ethics committee. Additional information regarding the ethical, cultural, and scientific considerations specific to inclusivity in global research is included in the Supporting Information.

## Results

### Bounding the systems

Three matrices were developed to synthesize findings from this research. Two matrices depict the high-level boundaries of the EYS system and health system in how they coordinate healthcare for YEH in Toronto, and one matrix depicts their combined efforts in coordinating healthcare for this youth population.

## An overview of the emergency youth shelter system

Within the EYS system, we found six key layers that affect healthcare coordination for YEH, as shown below in Table 3.

**Table 3:** Bounding the emergency youth shelter system.

System Layers	Niches	Organizations	Key Actors
Provincial government	**Policy Development** • Establish vision, legislative and policy framework for homelessness initiatives • Develop strategies, programs, and policies to measure, prevent, reduce, and end homelessness**Funding** • Fund homelessness programs and services • Establish national direction and negotiate federal contributions with federal government	Ontario Ministry of Municipal Affairs & Housing	Service managers: municipal service managers, and district social services administration boards
Local Government	**Administrative Tasks & Activities** • Administer funding to shelters • Develop shelter-based policies (e.g., Toronto Shelter Standards, harm reduction directive, etc.) • Plan and manage homelessness services • Provide relevant training to shelter providers and staff • Establish and manage central intake **Strategy & Program Development** • Develop strategies and programs to meet the needs of specific populations including youth	City of Toronto: Shelter, Support, and Housing Administration	City council, housing secretariate, general managers, policy development officers, housing commissioner of Toronto, etc.
Shelter providers (EYSs), including staff	**Deliver health promotion & health protection programs** • E.g., stress management programs, harm reduction programs, drug and alcohol awareness groups, art therapy, stay in school program, etc. **Provision of client-centred services/case management** • Work collaboratively with youth to develop a service plan that helps to achieve their goals • Provide services grounded in a harm reduction and trauma-informed approach • Identify needs beyond shelter and housing supports and work together with health services providers to facilitate access to other services **Collaboration, community engagement and partnerships** • Collaborate with clients, service providers and other stakeholders to create and maintain network of supports that helps youth achieve the best outcomes for themselves **Planned Discharges** • Ensure that youth have a discharge plan in place (e.g., to housing, treatment, hospital), whenever possible	E.g., Covenant House, Eva’s Initiatives for Homeless Youth, Youth Without Shelter, Horizons for Youth, Turning Point Youth Services, YMCA, Kennedy House Youth Services, Youthlink, etc.	Multidisciplinary teams include: Executive staff (e.g., executive directors, operations manager); Healthcare providers (e.g., physicians, counsellors, nurses, social workers, etc.); Frontline case workers (e.g., case managers); and Non-clinical frontline staff (e.g., intake coordinator, youth in transition workers, etc.).
Not-for-profit advisory body	**Collaborate through meetings** • Meet with other shelter providers, government, and partners to promote new methods designed to improve service delivery of youth shelter providers • Coordinate planning and service delivery for youth shelter providers **Advocacy** • Advocate for adequate funding to serve youth • Raise awareness about social and health programs run by youth shelters	Youth Shelter Interagency Network	Shelter providers
Community sector/non-government organizations	**Funding**Provide financial support to agencies that deliver essential services to help people move out of poverty**Research** • Create evidence to help communities prevent and end homelessness**Knowledge mobilization** • **Through the national conference, advocacy, and allied networks**	E.g., United Way Toronto Home Depot Foundation, etc. E.g., Canadian Observatory on Homelessness E.g. Canadian Alliance to End Homelessness	
Youth experiencing homelessness			

*Provincial & local government*. The provincial and local governments tackle homelessness more broadly through funding and high-level policy development [35]. While particular emphasis on youth homelessness within these levels is rare, their roles are critical in influencing subsequent system layers. First, the Ontario Ministry of Municipal Affairs and Housing lead the provincial government’s efforts to end homelessness, and is responsible for establishing the overall vision, legislative and policy frameworks for housing; developing strategies, policies, and programs to prevent, reduce and end homelessness; identifying desired outcomes and reports on their achievements; and working with the federal government to establish national directions and negotiate federal contributions towards Ontario’s housing and homelessness sector [35]. In Ontario, service managers are responsible for collaborating with frontline service delivery organizations including youth shelters to locally deliver housing and homelessness services [35].

The City of Toronto’s Shelter, Support and Housing Administration (SSHA) operates under the municipal government and directly operates some EYSs in the City, while also contracting community-based, not-for-profit agencies to provide emergency shelter services for youth [36]. The SSHA administers annual funding to shelters, develops shelter policies, provides relevant training to shelter providers, and manages the central intake of youth requiring shelter services [36, 37]. A City of Toronto executive elaborates: *“All of the youth services are delivered in partnership with our third party*, *non-profit organizations*, *but we’ve got a funding relationship with all of the agencies and are recognized by the provinces [as] sort of the service system manager for housing and homelessness services here in the city of Toronto*.*”* The SSHA also plays an important role in developing frameworks, strategies, and programs to meet the needs of specific populations experiencing homelessness, including healthcare programming for YEH. Nonetheless, there is evidence of poor engagement between the provincial and municipal levels of government within the sector [38, 39]. When asked about how to address funding challenges preventing the strengthening of healthcare coordination in the youth homelessness sector, the same City of Toronto executive explains:

*“It really depends on the province, right, because all of this [integration of health services] is provincially provided*. *You know, all the health care dollars, and we have taken, I’d say extraordinary measures during COVID to start directly funding mental health supports and some of the harm reduction supports. But it needs a long-term commitment from the province, and they have plans around ending chronic homelessness now by 2025- but these things need to be invested in and committed to.”*

*Emergency youth shelters*. At a macro level, shelter providers are responsible for supporting youth with achieving the best personal and health outcomes possible, by developing and maintaining networks of supports though collaboration with other social and healthcare providers [36]. Efforts to develop formal and informal partnerships to coordinate care within the broader health system, especially with hospital and community-based health organization staff, were clear through interviews with EYS executive and frontline staff. For example, a case manager at Shelter A shares, *“We’ve developed contacts within the hospitals that we can reach out to…I have a willing contact there who can connect with the youth and see if the youth will provide consent for us to figure out what’s been going on there*.*”*

At a micro level, EYS frontline staff work with their youth clients to develop personalized service plans through trauma-informed case management, and the delivery of various health promotion and health protection programs. They are also responsible for ensuring that their youth clients have a discharge plan in place whenever possible. Shelter providers along with their board of directors must comply with all relevant federal, provincial, and municipal legislation and regulations as stated in the Toronto Shelter Standards; in this case to support YEH with coordinating health services within and between the EYS and health systems [36].

*Advisory body*: *Youth shelter interagency network*. Further, the Youth Shelter Interagency Network; a not-for-profit community-based advisory group part of the larger Toronto Shelter Network, plays a significant role in working with the City of Toronto and advocating for the needs of youth residing within the shelter system [40]. The network, established in 1994, represents the youth sector and is comprised of Toronto-based EYS executives. The network raises awareness of the multi-faceted social and health programs run by Toronto shelters; coordinates the overall planning and service delivery for youth shelter providers; collaborates internally to resolve current and long-term issues within the system; and promotes new methods designed to improve service delivery within youth shelters [41]. The Director of Operations at Shelter A voices their sentiments on being involved in such a network:

“*The Youth Shelter Interagency Network gives us a platform to address the issue from a micro and macro perspective*. *These issues are brought forward*, *and the conversations are happening at a city level- so they’re on the ground*, *making sure that they come to fruition–so that’s a strength because that is a piece of the cooperative approach happening*.*”*

*Community-based*, *non-government organizations*. Moreover, several community-based, non-government organizations play a crucial role in funding services, conducting evidence-based research, and mobilizing knowledge to strengthen healthcare coordination for YEH. A few organizations known to have a personal stake in funding EYSs include the United Way Greater Toronto and Home Depot Foundation. Further, The Canadian Observatory on Homelessness is an example of a nationally leading research hub dedicated to ending homelessness in Canada, and has disseminated evidence on the need for systems integration and improved healthcare coordination within systems for YEH in Toronto [16]. Finally, The Canadian Alliance to End Homelessness (CAEH) is an example of another national organization leading the collaboration and movement of organizations, communities, and individuals to prevent and end homelessness in Canada. While not exclusively focused on the youth population suffering from homelessness, the CAEH promotes knowledge mobilization on research and programs related to YEH in Canada through their national conference including topics such as systems integration and healthcare coordination [42]. Another shelter executive explains how the Toronto Alliance to End Homelessness was a constructive outcome resulting from stakeholders’ convening at the annual CAEH conference:

*“The Toronto Alliance to End Homelessness started about six or seven years ago coming out of the national conference that we went to…and we realized we needed more coherent voices in Toronto that were going to*: *1) work effectively with funders and government, and a wide range of stakeholders but to also, 2) promote best practice both in terms of systems approach and coordinated access systems.”*

Since its conception, the Toronto Alliance to End Homelessness has been working collaboratively with other organizations and individuals to end homelessness in Toronto. For example, they have been hosting service planning forums almost monthly alongside the SSHA, City of Toronto; and Housing Secretariat to identify and discuss priorities for Toronto’s homelessness service system, including those affecting YEH [43].

*Youth with lived experience of homelessness*. Finally, the EYS system centres around serving the needs of YEH. Capturing their unique experiences navigating both systems when in need of clinical healthcare is pivotal to understand strengths and gaps within both systems. Therefore, YEH make up a key system layer across the EYS and health systems, as shown in the following two matrices as well. Overall, youth have autonomy over their health and healthcare and may choose to access care at various organizations that offer health services within any of the bounded systems.

### Collaboration and overlap between the EYS and health systems

The following matrix presents collaborative efforts and overlap between organizations and actors within the EYS and health systems, who provide health services to YEH and/or support them with accessing coordinated care in Toronto. (Table 4)

**Table 4:** Collaboration and overlap between the emergency youth shelter & health systems.

System Layers	Niches	Organizations	Key Actors
Provincial government	**Provision of integrated and continuous healthcare services** • Healthcare through Ontario Health Teams • Mental health, substance use, primary care, system navigation services through Youth Wellness Hub (E.g., mental health counselling, primary care, psychiatric consultation, care navigation, youth drop-ins, peer support group, etc.)	Ministry of Health Ontario Health Youth Wellness Hubs Ontario (Toronto Central and Toronto East)	**Ontario Health Teams**—All stakeholders involved in: Primary care, acute care, mental health and addictions, community care, long-term care. **Youth Wellness Hubs**: Hospitals (e.g., Sick Kids Hospital, CAMH, etc.); Community services (e.g., Loft Community Services, Lumenus, etc.); City of Toronto–Toronto Youth Partnership and Employment; The Sashbear Foundation; Strides, Vibrant Healthcare Alliance
Local Government	**Support with establishing programs for coordinated access to healthcare** • Coordinated Access to Care from Hospital (CATCH)- Homeless Program through Inner City Health Associates • Coordinated access to mental health and addiction services and supportive housing through the Access Point	Inner City Health Associates Toronto Mental Health and Addictions Access Point (Access Point)	**CATCH-Homeless program**: St. Michael’s Hospital, Toronto North Support Services, transitional case managers, healthcare providers, community-based organizations **Access Points**: Hospitals and community service organizations
Not-for-profit healthcare centres for Homeless Youth	**Provision of care through clinical programs** • Clinical drop-in programs • Dental programs • Mental health care and counselling **Referring youth to primary care at hospitals**	• Youth Street Mission LOFT Community Services	Volunteer healthcare providers (e.g., physicians, dentists, counsellors, etc.)
Private Sector	**Integrated clinical care at emergency youth shelters** • E.g., Cognitive Behavioural Therapy, Dialectical behaviour Therapy Case management and referrals to external healthcare from shelters	Private Organizations (e.g., Allied Health Services, Centre for Cognitive Behaviour Therapy)	Healthcare providers, including psychiatric associates and consultants
Working Groups	**Toronto Shelter Network working group** • Strengthen communication and engagement between system stakeholders Establishment of **Shelter Health Services Advisory Committee** • Developed coordinated approach to health services delivery for shelter clients	Toronto Shelter Network City of Toronto & Local Health Integration Networks	• Shelter providers, City of Toronto, and other agencies Shelter providers, shelter clients, and local health service providers
Youth experiencing homelessness			

*Provincial government*: *Youth wellness hubs & Ontario health*. In 2017, the provincial government announced funding for the establishment of Youth Wellness Hubs across Ontario to address gaps in the youth service system. This included access to mental health, substance use, primary care, housing, and other community-based and social services for youth between 12 and 25 years of age. Youth Wellness Hubs also include system navigation services that are emphasized to be “timely, integrated and co-located.” An executive at Shelter B shares their thoughts on the rise of these newly developed hubs:

*“Youth in crisis, in shelter, need health services–connection to specialized health services particularly for young people is very, very critical*. *I think the Youth Wellness Hubs that the province has been developing is a positive step in that direction.”*

Currently, there are 12 hubs across the province that offer services to youth in need, including the Central Toronto and Toronto East Hubs, which began operating in 2017 and 2019, respectively [44].

Further, the provincial government legislated Ontario Health in 2019 through the Connecting Care Act; one single health agency overseeing the provision of integrated, continuous, and coordinated care through Ontario Health Teams (OHTs). OHTs comprise of partnerships between organizations and/or service providers to serve populations concentrated in various regions within the province [45, 46]. Organizations and/or service providers within each team should be able to deliver at least three of the following services to their designated population: primary care, emergency health services, mental health and addictions services, home care or community services, long-term care, palliative care, rehabilitation and complex care, and/or other prescribed health or non-health services supporting healthcare provision [46]. OHTs continue to form across Toronto and include some EYS and healthcare service providers. A shelter executive alludes to their involvement in an OHT as having the potential to support YEH with improved access to health services:

*“A lot of [Shelter A’s] revenue comes from donations from corporations. So, we have not been a traditional health funded organization, right*. *[But], we are a member of the Downtown East Ontario Health Team.”*

*Partnerships*: *Public sector*, *not-for-profit and private sector organizations*. Aside from government legislated initiatives to strengthen healthcare coordination, EYS leadership have established both formal and informal partnerships with the Ministry of Health and/or City of Toronto funded, not-for-profit, and/or private organizations to provide health services in-house or externally through referral to clinics, hospitals, or community-based organizations in their respective regions. Each EYS has a formal partnership with the Inner City Health Associates (ICHA), where physicians, nurses, and/or psychiatrists come to their designated shelter on a weekly basis to provide healthcare for youth [47]. ICHA also offers programs such as Coordinated Access to Care from Hospital (CATCH)- Homeless, which was created in collaboration with St. Michael’s Hospital and the Toronto North Support Services, to help people experiencing homelessness who have unmet and complex healthcare needs access appropriate health resources in their community. CATCH transitional case managers at partner hospitals refer clients experiencing homelessness to the program, and work with healthcare providers to support these clients’ including youth, access medical, psychiatric and addictions services [48].

Another example of publicly funded efforts to integrate shelter and health services are those through the Toronto Mental Health and Addictions Access Point. The Access Point provides youth who are 14 years of age or older, and who suffer from mental health and/or addictions with intensive case management; and access to assertive community treatment teams, and programs including mental health, supportive housing, and problematic substance use housing [49]. In describing their pathway to care, a young person experiencing homelessness who suffered from complex psychiatric comorbidities and addictions explains:

*“When I left treatment [at Homewood Health], I did an Access Point application on the request of my treatment team. I think when I got home, that was probably the most useful thing that I did in 2017*… *through Access Point, I got a LOFT caseworker. I applied for case management, the caseworker worked for the addictions stream through the LOFT-A hub, and got me set up with psychiatrist, and then helped me get referred to the COMPASS program at CAMH, which is for people on medically assisted opioid withdrawal.”*

Access Point partners include various hospitals and community service organizations. However, to date, there are no EYSs listed as Access Point partners [50]. As demonstrated by one of the youth’s experiences above, partnerships between EYSs and Access Points may support YEH receive the specialized healthcare and housing they need for their mental health and addictions concerns or comorbidities.

Finally, private organizations are sometimes contracted by shelter providers to provide healthcare on site, such as counselling, and other mental health and addictions supports for youth in need. External referrals by contracted healthcare providers or EYS frontline staff are also sometimes initiated for youth to receive primary or specialized healthcare outside the shelter system. Moreover, youth are sometimes referred to not-for-profit, community-based organizations such as the Evergreen Health Centre at the Youth Street Mission, to receive care such as STI testing, dental care, and mental health counselling.

*Working groups*. As part of their strategic plan for 2020–2023, the Toronto Shelter Network aimed to strengthen communication and engagement between their diverse group of member agencies including the many EYSs in the City. One way through which this was galvanized was the formation of working groups, in which member agencies identify and pursue shared goals [51]. For example, an executive at Shelter C, who is involved in the network shares how one of the working groups they are currently involved in within the network focuses on EYS engagement with the broader health system. They share that the challenge with such engagement is, “*…quite honestly*, *healthcare and shelter*, *support*, *and housing—they don’t connect*.”

Furthermore, the Shelter Health Services Advisory Committee, which used to consist of the Toronto Central and Central East local health integration networks, the City of Toronto, and health services and shelter providers, is another example of a working group established to improve equity and access to health services for shelter clients, specifically through a coordinated approach to healthcare [52]. Unfortunately, this 2017 initiative was paused shortly after its conception due to the newly elected provincial government (2019) planning many structural changes within the health system, and the COVID-19 pandemic burdening both systems in 2020.

### Health system role in healthcare coordination

Organizations and actors within the health system are significantly less involved than those within the EYS system in their efforts to coordinate healthcare for YEH in Toronto. This is presumably due to their provincial and local mandates to serve their designated regional population(s) as opposed to focusing exclusively on YEH [53]. Nonetheless, elements of the system that are involved in healthcare coordination are depicted below in Table 5. As the federal and provincial governments do not focus exclusively on healthcare coordination for this population, they have been excluded from this section.

**Table 5:** Health system role in healthcare coordination.

System Layers	Niches	Organizations	Key Actors
Local Government	**Health policy, programming, and service delivery:** • Data collection to inform health service delivery improvements for homeless populations • Delivery of health services and programs (e.g., youth sexual health, alcohol, and other drugs, etc.) • Development of public policy and practices	City of Toronto: Toronto Public Health, The Works, Toronto Board of Health, City council	Local medical officer of health, healthcare providers, policy makers, health promoters
Primary care, including community care	**Health promotion programs at community health centres** • E.g., Urban Health, Take home Naloxone, Hep C, and Health Bus programs at Sherbourne Health **Clinical programs at community health centres** • Drop-in primary care services **Virtual mental health care** • E.g., What’s Up Walk-in clinics	Hospitals, walk-in-clinics, community health centres, Toronto Public Health	Primary healthcare teams, family health teams, allied health professionals, youth mental health workers, health promoters, addiction workers, etc.
Emergency services	**Emergency care at hospitals during crises** • Crises services and mental health emergency at hospital emergency departments **Pilot programs through hospital research centres** • E.g., YouthCan IMPACT at CAMH, and the Navigator program and Phone Connect program at St. Michael’s Hospital	Hospitals such as Centre for Addiction and Mental Health, those through Unity Health, University Health Network, etc.	Paramedics, emergency physicians, nurses, personal support workers, etc. health navigators, outreach counsellors
Specialty care	**Specialized care at hospitals or in the community** • Services coordinated to provide continuity of care • Mental healthcare, addiction services • Long-term care	Hospitals, community and social service organizations	Professionals who provide services partially funded or privately insured services (E.g., pharmacists, mental health counsellors, etc.)
Private health services	**Provision of private healthcare services, which include:** • Dental care • Vision care • Complementary and Alternative Medicine • Outpatient Physiotherapy	Privately owned clinics and healthcare professionals operating privately (e.g., Covenant House partnered with Accenture Dental).	Healthcare providers including dentists, optometrists, physiotherapists, etc.
Youth experiencing homelessness			

*Local government*: *City of Toronto*. Toronto Public Health promotes the health of residents through the delivery of health services, the development and implementation of healthy public policy and practices, and data collection to help inform how best to meet community needs. Toronto Public Health reports to the Toronto Board of Health, who ensures that programs and services are delivered adequately based on Ontario’s standards and in response to the population’s local needs [54]. Although programs and services are offered based on broader population categories, several are relevant to the needs of YEH, including: support for people seeking treatment for substance use, tobacco prevention for youth, and sexual health counselling [55]. In June 2021, the City of Toronto issued a directive to shelter providers to implement harm reduction policies and procedures on-site. A City of Toronto executive shares their perspective on why the implementation of such an initiative might be impactful:

*“…in order to address the broad determinants of health that keep people housed*, *there’s a significant component related to accessing primary health care*, *mental health supports*, *harm reduction supports*, *and housing*, *[and] they all kind of need to be packaged together… I think that we’re getting there*. *More recently with the city’s 10-year plan on housing*, *one of the core components is recognizing that we need a lot more supportive housing in the city to allow people to move out of homelessness*, *and [ensure] they keep their housing and part of the supports that are needed in that housing*, *again relate to the integration of those health supports [on-site] to ensure that people are successful*.”

The 10-point plan developed by The Works at Toronto Public Health is intended to help shelter providers implement best practices in providing harm reduction supports on site. The plan emphasizes ten areas of harm reduction programming that should be established at 24-hour homelessness service centres. Some of these areas include the availability of harm reduction supplies on-site, training for staff on the harm reduction approach, the implementation of harm reduction and drug use policy, and implementation of overdose prevention and response interventions [56]. The Works has offered harm reduction supplies and services to over 100 locations and Access Points in Toronto, including EYSs such as Eva’s Satellite and the YMCA [57].

*Primary healthcare services*. Primary healthcare services most needed for YEH include: routine care, care for urgent but minor or common health issues, mental healthcare, psychosocial services, health promotion and disease prevention [58]. Executive and/or frontline staff at Toronto-based EYSs either refer youth to primary care providing centres or establish formal or informal partnerships with them. These services are typically provided by family physicians, nurse practitioners, or general practitioners as the first point of contact for medical care within the health system [58]. A case manager at EYS A discusses their perspective on preferred avenues for primary care for YEH:

*“We’re located in an area where there’s a hub of social and health services*…*and so obviously we know that more community forms of health care [and] primary care, that are available, the less likely they [YEH] will enter the emergency departments. I think in a lot of cases, if we have staff members that are not medically trained, and if healthcare is closed. In general, like when you have a young person who’s chronically experiencing like passive suicidality, [then] we send them to the hospital.”*

This quote exemplifies the need for EYS and community-based primary healthcare partnerships to appropriately coordinate care for YEH and prevent them from possible entry into the endless shelter-hospital loop. Other examples of primary care accessed by youth include free virtual mental health counselling through What’s Up Walk-in-Clinics, and drop-in or scheduled clinical or health promotional services at nearby community-based agencies (e.g. health centres).

*Hospitals*: *Emergency services & research*. During health crises, paramedics are often called to transport youth from the shelter they are staying at to the nearest hospital emergency department; this includes the mental health emergency department at the Centre for Addiction and Mental Health. Additionally, various pilot programs which focus on improving healthcare coordination for YEH and other homeless populations are run by researchers through hospital-based research centres. One example is that of the YouthCan IMPACT trial at the Centre for Addiction and Mental Health, through which an integrated community-based collaborative care team model was implemented and evaluated to bring together a wide range of supports in one location for the mental health, substance use, and wellness needs of Toronto-based youth [59, 60]. The navigator program at St. Michael’s Hospital MAP Centre for Urban Health is another example where homelessness outreach counsellors and health navigators aid people experiencing homelessness who come to the emergency department navigate continuity of care for their health and social needs. This is achieved by communicating with emergency shelter and community agency staff to ensure they have access to the necessary health information, medication, supplies, and personal care to support client [61]. Further, if specialty care is required based on healthcare providers’ assessments at the hospital, youth may be appropriately referred to resources on-site. For example, an emergency physician at a Toronto-based hospital explains:

*“We see a number of our mental health patients are often struggling with housing*. *And so often they’re directed to the mental health youth in crisis worker, who [then] takes over the process.”*

*Referral to specialized services & private care*. Both primary healthcare and emergency care providers refer patients to specialized care as needed based on their clinical assessments. Depending on the type of specialized care required, this second-level of care is typically accessed at higher levels of hospitals or in the community. Some specialty services are fully or partially covered by the Ontario Health Insurance Plan, such as eligible dental surgery in hospital, eligible optometry, abortion services with a prescription from a doctor, and podiatry [62]. Private health services such as dental and vision care are significantly less accessible than publicly funded services for youth who have an Ontario Health Insurance Plan. In some cases, shelters are partnered with privately run dental clinics to help youth receive access to this care. Overall, access to specialty care is quite limited for this population, as patient socioeconomic status is found to be associated with poorer access to specialist care [63, 64].

### Tying it altogether: Inter-organizational collaboration & connections between systems

**Fig 1:**
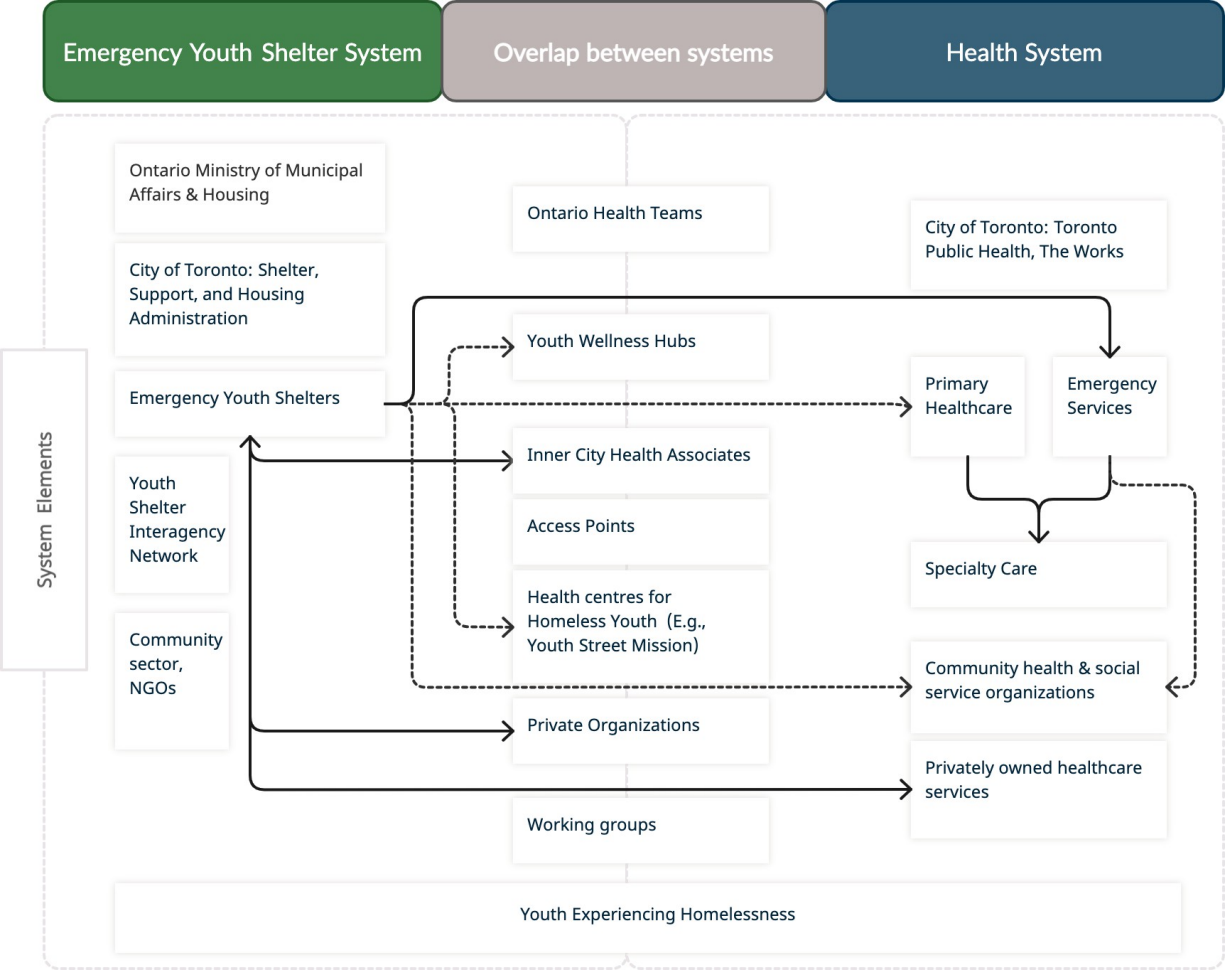
Connections between elements within bounded systems.

The following diagram illustrates connections between system elements confined within the boundaries of the Toronto-based EYS system, health system, and those that overlap between both systems, specifically in their healthcare coordination roles for YEH (see Fig 1). Arrows between systems illustrate pathways to healthcare as coordinated by executive or frontline staff within the defined boundaries of each system.

Findings from this research demonstrate that support with coordinating healthcare for and with YEH is most initiated by EYS leadership and staff. Youth may access health services that are integrated on-site through ICHA, or through other shelter-health system partnerships developed by EYS executive and/or frontline staff. These include partnerships with external institutions, organizations, and/or clinicians who provide health services and are depicted by the solid, bidirectional arrows. Youth may also be referred to these institutions or organizations by frontline staff within either system based on their knowledge of health services provided by these external groups, which is depicted by the dashed, unidirectional arrows. Finally, youth may be sent to the hospital emergency department and/or subsequent specialty care by system staff when facing health crises or in need of urgent care, and this is depicted by the solid, unidirectional arrows.

## Discussion

In this study, we define the boundaries of the EYS and health systems, specifically in their roles coordinating healthcare for YEH. By identifying relevant levels, niches, organizations, and actors that lie within each system and understanding how they interact, we are better positioned to think about successive research, helpful partnerships within and between each system, and system-level interventions that might be worth implementing and evaluating. While it is well known that the EYS and health systems operate largely in silos, this research has demonstrated collaboration and overlap between both systems in their efforts to improve care coordination through various initiatives led by public, private, and not-for-profit organizations. While efforts to strengthen healthcare coordination are evident by stakeholders within the EYS and health systems independently (e.g., through the development of formal and informal partnerships between vested organizations and actors), there are several barriers that have made this an ultra-challenging task, including the complex nature and historically siloed structures of these two systems and neither sector being mandated to coordinate healthcare for this population. Findings from this research also indicate that efforts to coordinate healthcare within and between system boundaries are largely influenced by government funding (macro level), availability and awareness of relevant organizations and services offered across systems (meso-level), and prioritization of research and funding based on the interests of vested stakeholders (micro level).

At a macro-level, the high-level roles (e.g. policy development, funding, etc.) and priorities of various government bodies significantly impact healthcare coordination processes and outcomes within and between the EYS and health systems. Each of these systems are funded by and operate under two different levels of government, causing several inconsistencies between sectoral parts; this increases complexity in care coordination pathways, and therefore youths’ healthcare coordination outcomes [22]. For example, hospitals which are provincially funded and shelters which are municipally funded; are two critical elements of each system that are extensively accessed by YEH but do not homogenously offer health or health coordination services across systems. This is especially true for the EYS system where the City of Toronto partially funds shelter providers based on their capacity to provide services, while the rest is expected to be fundraised [41]. The difference in funding provided by the City to youth shelters, and various shelters’ capacity to fundraise is one reason why Toronto-based EYSs have inconsistencies in the programs and services they offer to youth [65]. An EYS with a robust fundraising program, is more likely to offer either a wider range and/or more specialized health services for youth clients. For example, Eva’s Satellite in Toronto is unique in that it offers harm reduction and addictions support for youth who are struggling with substances [66], while Covenant House has an integrated health centre where youth can access a range of primary care and mental health supports [67].

At a meso-level, there are numerous organizations and actors that provide healthcare services across and between each system either for the general population or targeted to the needs of YEH (e.g. Evergreen Health Centre). In this study, we learned that EYS staff prefer to refer their youth clients to external community-based services over sending them to public hospitals if youths’ required healthcare needs cannot be met on-site. This preference of community-based care referrals may be due to the reduced likelihood of youths’ entrapment in the vicious shelter-hospital loop [15]. However, a recent study suggests that community-based health services targeted to meet the unique needs of people experiencing homelessness including youth are scarce and inequitably distributed in Toronto making it additionally challenging for EYS staff to coordinate external community-based care for their youth clients, or for hospital staff to coordinate this care post-hospital discharge [22]. Further, it was reported that many staff within both sectors have limited knowledge and awareness of community-based services that are available to appropriately support YEH’s distinct healthcare needs. However, as mental health and addictions services are most critically needed for YEH, it is evident that efforts are being made by decision-makers to increase accessibility, provision and coordination of mental healthcare for YEH [1, 10]. This is seen through the establishment of Youth Wellness Hubs, What’s Up Walk-in Clinics, and Access Points. Furthermore, while there was mention of one of the EYS’s involvement in a newly established OHT, we did not find any reports or evidence on care coordination processes or outputs related to the OHT serving YEH.

At a micro-level, healthcare coordination for YEH depends significantly on the interests and priorities of independent agencies and vested stakeholders such as researchers and funders. While research plays an important role in understanding healthcare coordination between sectors; and recommending, developing, and evaluating interventions to strengthen coordination, they are often limited to the restraints of researchers’ interests, funding they receive from vested third-party organizations, and their personal timelines. Some examples of promising initiatives to improve healthcare coordination between systems in Toronto include the St. Michael’s hospital Navigator program [61], the At-Home/Chez-Soi randomized control trial of Housing First intervention [68], and various peer-support models that have been piloted within systems [69]. One example of a national program that has successfully supported healthcare coordination for homeless populations and have tackled some of the macro, meso, and micro-level challenges discussed is the US Healthcare for the Homeless (HCH) program.

The HCH programs are part of community-based organizations that provide low or no-cost healthcare to people experiencing homelessness and are funded and regulated through the Health Resources and Services Administration at the US Department of Health and Human Services [70]. In the HCH model, the US healthcare system emphasizes a multidisciplinary approach to care coordination by collaborating with community-based health and social service agencies. The model relies heavily on on-site case managers to advocate for their clients, and link them to appropriate community-based health and social service resources, ensuring that a wide range of their health needs are being met [71]. Other research and program evaluations have shown that employing staff such as navigators, youth workers, case managers, and peers who have lived experience of homelessness, within either system can play an important role in helping people experiencing homelessness transition between systems and receive the necessary follow-up and post-discharge care [69, 72, 73]. Overall, establishing inter-organizational staff roles that focus on providing navigational, transitional, and/or peer support have shown to be promising in strengthening healthcare coordination for YEH within these complex and fragmented systems, as seen with the HCH model.

### Strengths and limitations

A major strength of this study is the triangulation of documents and key informant interview data to respond to the research questions [74]. A potential limitation to the study is the non-exhaustive nature of the document analysis, which may suggest biased selectivity [75]. While a wide range of documents were analyzed to provide broad coverage, not all organizations that fall within each system layer were explored in detail. Nonetheless, a high-level depiction of how each system is bound was possible with the methodology used to retrieve documents included in this study. Second, few relevant scholarly articles were found for inclusion in this study, and the reliability of some grey literature was uncertain. For example, it is possible that some organizational websites are not updated on a regular basis. Another limitation to the study is the small number of key informants recruited from the large and complex health system. Despite our repeated attempts to recruit health system staff, only a few healthcare providers were responsive or available for an interview. The low response rate was largely due to the health system burden caused by the COVID-19 pandemic, which was at a peak during the study period.

### Future directions

Based on the framework for transformative systems change, this study is the first of three steps required to identify interventions or “levers for change” to strengthen healthcare coordination within and between the EYS and health systems for YEH in Toronto [25]. The aim of the larger case study is to share tangible ways in which to improve healthcare coordination for YEH, alongside stakeholders who work at various levels within each system and intersectorally. The subsequent articles in this series explore: 1) pathways to healthcare taken by YEH who reside at EYSs in Toronto, as well as factors that influence these pathways to care [15], 2) causal links between key systems variables and resulting feedback loops, which illustrate relationships and interdependencies within and between these two systems [20], and finally 3) interventions that should be considered to strengthen healthcare coordination for YEH, both within and between the EYS and health systems in Toronto.

## Conclusion

Findings from this qualitative study reveal a vast network of organizations and actors that fall within each system layer, and work both in silos and collaboratively to coordinate health services for YEH. The EYS system was found to play a more active role in these efforts, while layers of the health system tend to have similar protocols for YEH as they do for the general population. There are, however, some organizations within the health system that are mandated to serve this specific youth population, in addition to efforts initiated by vested stakeholders within the system to improve access, quality, and coordination of healthcare for YEH. For the past few years, several organizations, and actors across various layers of both systems, have been collaborating to better coordinate healthcare for YEH, with the end goal of improving health and housing outcomes, and thereby preventing and/or ending chronic youth homelessness.

## Supporting information

S1 File Detailing documents considered for document analysis. (DOCX)

S2 File Approval certificate. (PDF)

## Abbreviations

YEH: Youth experiencing homelessness
EYS: Emergency youth shelter
SSHA: Shelter, Support & Housing Administration
ICHA: Inner City Health Associates
CAEH: Canadian Alliance to End Homelessness
OHT: Ontario Health Team
CATCH: Coordinated Access to Care from Hospita
HCH: Healthcare for the Homeless
