# Supplementary material for: Bounding systems: A qualitative study exploring healthcare coordination between the emergency youth shelter system and health system in Toronto, Canada
Source: PLoS One. 2024 Jun 21;19(6):e0303655. doi: 10.1371/journal.pone.0303655 (PMC11192382; doi:10.1371/journal.pone.0303655)
Supplement: S1 File — (DOCX) [file pone.0303655.s001.docx]

## Table 1: Documents analyzed for inclusion in document analysis

| **Document type** | **Document name** | **Organization** | **Retrieval Site** | **Summary/Notes** |
| --- | --- | --- | --- | --- |
| **WEBSITES** |  |  |  |  |
| 1 | Covenant House - Health & Well-being support programs | Covenant House | https://covenanthousetoronto.ca/our-solution/health-and-well-being/ | Health programming seems to be very holistic in comparison to other youth shelters. This may be the case due to the funding capacity that Covenant House has. Also, many strong external partnerships listed here for provision of health services (health promotion and health care). |
| 2 | Eva's Initiatives for Homeless Youth - Harm Reduction | Eva's Initiative for Homeless Youth | https://www.evas.ca/what-we-do/harm-reduction/ | Overarching charity with three main shelters sites (2 EYSs and 1 transitional). Have included Youth Belong program as key component of all three shelters. Harm reductions supports emphasized in health programming. |
| 3 | Eva’s Satellite | Eva's Initiative for Homeless Youth | https://www.evas.ca/where-we-are/evas-satellite/ | Specialized harm reduction shelter for youth aged 16-24. Partnered with ICHA, Central Toronto Youth Services, and other community agencies.  ICHA providers psychiatric, primary, and preventative health services on site twice per week. Also have a sexual health clinic which runs every 2 months. |
| 4 | Eva’s Place | Eva's Initiative for Homeless Youth | https://www.evas.ca/where-we-are/evas-place/ | Partnered with New Outlook and East Metro Youth Services to provide counselling and referral services. Also partnered with ICHA who provide primary and psychiatric care. |
| 5 | YWS - Emergency Residential Program | Youth Without Shelter | https://yws.on.ca/how-we-help/emergency-residential/ | There is no health and well-being specific page. Information is broadly stated (2021). This has now been updated to include mental health programming (2023). |
| 6 | YWS: Mental health program | Youth Without Shelter | https://yws.on.ca/how-we-help/mental-health-program/ | Launched in 2021 and includes individual psychotherapy, diagnostic and psychosocial assessments, ODSP applications support, connecting youth with other external resources. |
| 7 | Kennedy House – overview of services | Kennedy House Youth Services | https://kennedyhouse.org/what-we-offer/ | Health services not explicitly listed here. |
| 8 | Horizons for Youth – mental health and wellness support | Horizons for Youth | https://horizonsforyouth.org/mentalhealth | Various levels of mental health supports described, including community referrals to outpatient programs at hospitals, support groups, and community health centres. |
| 9 | Turning Point Youth Services – Our programs | Turning Point Youth Services | https://turningpoint.ca/programs/ | Focus on counselling for individuals, family, and group. Age is defined as 12-17 years old for eligibility for services without charge. |
| 10 | Building a connected public health care system for the patient | Ministry of Health and Long-Term Care | https://news.ontario.ca/en/backgrounder/51360/building-a-connected-public-health-care-system-for-the-patient | Describes new model of integrated care through Ontario Health and the establishment of OHTs |
| 11 | Ontario Health Agency | Ontario Health | https://www.ontario.ca/page/ontario-health-agency | Describes Ontario Health as one single agency preparing to oversee healthcare delivery, improve clinical guidance and provide support for providers to ensure better quality of care for patients. Web page outlines responsibilities of the agency and overall changes made to the system because of its establishment. |
| 12 | Providing CARE to help the homeless | Arlene Howells, Unity Health Toronto | https://unityhealth.to/2020/10/providing-care-to-help-the-homeless-in-toronto/ | Quote: Reflecting on the various forms of support she provided to ICHA, Dr. Robertson notes that “Hospitals need to be more proactive to cultivate and establish important relationships with community support organizations such as shelters. As we are continuing to learn, hospitals need to expand their role to support public health directives in the pandemic.” Collaborating with groups like ICHA can help reduce the number of people who end up in a hospital emergency department for treatment during the pandemic and more generally reduce the harms caused by the pandemic.  Interesting to note, as it seems that most of the ownness through other documents on relationships development seems to be falling on shelter operators. Interview with doctor also makes it seem that this falls outside of scope of hospital with the capacity they have (assume this includes EYSs) |
| 13 | Sherbourne Health – urban health programs | Sherbourne Health | https://sherbourne.on.ca/primary-and-family-health-care/priority-populations/urban-health/ | Description of Health bus program, HEP C program, Take home naloxone program, and WINK program for women. Links to more information for each program is provided on web page. |
| 14 | Providing health services for people experiencing homelessness in Toronto | Inner City Health Associates | https://www.icha-toronto.ca/ | Describes health care programs provided by ICHA to homeless populations across Toronto: transitional primary care, psychiatric care, and palliative care. ICHA is funded by Ontario Ministry of Health, and works with City of Toronto, hospitals, and community health and social support organizations including youth shelters. |
| 15 | Welcome to the Toronto CATCH program | Inner City Health Associates | https://www.icha-toronto.ca/programs/welcome-to-the-toronto-catch-program | Described as a service for those who are experiencing homelessness and are not connected to services, with or without mental health or addiction problems. It is a collaboration between ICHA, St. Michael's Hospital and Toronto North Support Services. Page includes toll free number, CATCH partners, and a copy of the referral form for download.  - Not youth-specific. |
| 16 | The Access Point: About | The Toronto Mental health and addictions access points (The Access Point) | https://theaccesspoint.ca/about/ | Centralized to provide and connect individuals above 14 years of age with mental health and addictions support services and supportive housing.  Some mention of partnership with Shelter B executive, but no mention of any formal partnerships on Access Point website. |
| 17 | MAP Centre for Urban health solutions - Navigator project | St. Michael’s Hospital | https://maphealth.ca/navigator/ | Pilot project employing homeless outreach counsellors to help people experiencing homelessness navigate health and social supports post- hospital discharge. Not youth-specific, but relevant in efforts to improve healthcare coordination within and between systems. |
| 18 | Counselling and Psychotherapy | Youth Link | https://youthlink.ca/services/our-programs/counselling/ | A multi-service agency providing emergency shelter and other community-based programs for YEH, including counselling and psychotherapy. Services include live-in treatment at Constance House, ongoing counselling, and what’s up walk-in.  It is one of the first shelters to follow the ‘new shelter model.’ It was formerly the Big Sisters of Metropolitan Toronto and is now an EYS and transitional shelter with embedded mental health agency. |
| 19 | Need to Talk? | What’s up Walk-in | https://www.whatsupwalkin.ca/ | Free in-person and virtual mental health counselling for children, youth, young adults, and families – offered as a resource at some EYSs in Toronto. |
| 20 | Central intake | City of Toronto | https://www.toronto.ca/community-people/housing-shelter/homeless-help/central-intake/ | Services and process information about the City of Toronto’s centralized intake line, used to connect people experiencing homelessness (including YEH) with information about EYSs with available beds.  Also facilitate warm transfers to specialized services such as primary and mental health supports. ** |
| 21 | Canada’s healthcare system | Government of Canada | https://www.canada.ca/en/health-canada/services/health-care-system/reports-publications/health-care-system/canada.html | Introduction, background, and role of different levels government in Canada's healthcare system. |
| 22 | Youth Wellness Hubs Ontario: Central Toronto (services) | Youth Wellness Hubs | https://youthhubs.ca/en/sites/central-toronto/ | Funded by the province in 2017 – integrated service hubs for Youth to address gaps in youth service systems. YWHs are one-stop-shops for youth aged 12-25 to address their needs including housing and health needs. Include outreach, peer services and system navigation services. Purpose is for services to be timely, integrated and co-located. |
| 23 | Youth Healthcare - Evergreen Health Centre | Yonge Street Mission | https://www.ysm.ca/get-help/health-care/ | YSM is collaborative partner with St. Michael's Hospital, contributing as a partner in their Family Health Team (from brochure). They help to connect youth aged 16-24 experiencing homelessness to primary care. The Evergreen Health Centre provides a range of public and privates services to YEH at no cost. |
| 24 | TSN Sector Tables | Toronto Shelter Network | https://www.torontoshelternetwork.com/sector | Describes various TSN sector tables – describes layers in advisory and working groups. |
| **REPORTS** |  |  |  |  |
| 25 | System in Crisis: An action plan for the future of Toronto's Homeless Youth | Youth Shelter Interagency Network | https://www.toronto.ca/legdocs/mmis/2007/cd/bgrd/backgroundfile-2777.pdf | Strong tone - urgent and angry  - last report found through City Council is from 2007  - 11 youth shelters at the time - this has changed now. Different folks also likely on YSIN.  - request for provincial government action plan, and integration of health services within shelters |
| 26 | 500 in five: Strategic Plan 2019-2024 | Youth Without Shelter | https://yws.on.ca/wp-content/uploads/YWS_18002_StrategicPlan_Public_FNL_WEB.pdf | Outlines six principles that guide the 2019 -2024 strategic plan; and 3 key strategic area goals with rationale, including: providing quality wrap- around supports for youth living at YWS; providing quality transition and after-care support; and education, awareness and advocacy on issues directly related to the youth YWS serves. Mission support goals and rationale also provided. |
| 27 | COVID-19 Interim Shelter Recovery Strategy: Advice from the homelessness service system | Prepared by BGM strategy group for: City of Toronto and United Way Greater Toronto | https://www.toronto.ca/legdocs/mmis/2020/ph/bgrd/backgroundfile-156419.pdf | The report offers advice to guide the City of Toronto - SSHA, United Way Greater Toronto, agencies, and other partners in responding to the COVID-19 pandemic in the shelter and homelessness service systems. A foundation for long-term collaboration on best practices, policy, and strategic investments to end homelessness is offered in the report, including specific actions to deepen collaboration and coordination between emergency shelter system and health partners.  Included because of the collaboration ("tireless commitment, partnership and strong communication across homelessness and health sectors.")  - implementation of the coordinated health services for shelter client’s framework recommended  - Coordinating safe supply of medications and other harm reduction supports for individuals who use substances was an area recognized to need greater coordination between different levels of government |
| 28 | Youth at the Centre of Impact: Towards an outcomes measurement framework | Prepared by: Dr. John Ecker- Director of Evaluation at COH, Jesse Donaldson- Deputy Chief Operating Officer at COH, Jocelyn Helland - ED at Eva's | https://www.homelesshub.ca/sites/default/files/attachments/outcomes.measurement.report.15nov17_0.pdf | Living document - several key organization and actors mentioned in detail in this report.  - focus is more on Eva's and less so on any coordination of health services with the health system. Physical and mental well-being (including accessing health services) are one of the main outcomes of mastery and independence, which falls under youth development outcomes. |
| 29 | Engagement Framework | City of Toronto (Shelter, Support and Housing Administration) | https://www.toronto.ca/wp-content/uploads/2017/10/971f-SSH-Engagement-Framework.pdf | Outlines the vision, principles, commitments, and definition of engagement by the SSHA in supporting a more integrated, and client-centered housing stability service system. |
| 30 | Harm Reduction Framework: Fostering dignity for people who use substances across and housing and homelessness services | City of Toronto (SSHA) | https://www.toronto.ca/wp-content/uploads/2017/10/9791-SSHA-Harm-Reduction-Framework.pdf | Framework developed through research focused on emergency shelter system - key informant interviews and literature review with stakeholders including SSHA and TPH staff, community health sector, people with lived experience, and shelter and social housing providers. |
| 31 | Homelessness Solutions Service Plan | City of Toronto (SSHA) | https://www.toronto.ca/legdocs/mmis/2021/ec/bgrd/backgroundfile-171730.pdf | Outlines implementation priorities for the next 3 years, using an integrated and person-centered approach to address homelessness. Developing an integrated systems response is highlighted as one of the priorities in this plan. The plan also provides context on the role of SSHA in service delivery and how the homelessness service sector operates within broader housing system in Toronto. |
| 32 | Ontario's Housing and Homelessness System | Province of Ontario | https://www.ontario.ca/document/community-housing-renewal-ontarios-action-plan-under-national-housing-strategy/ontarios-housing-and-homelessness-system | Various chapters to scroll through - provides summary of government role in the housing and homelessness system, key actors involved in executing key strategies. |
| **POLICY DOCUMENTS** |  |  |  |  |
| 33 | People’s healthcare Act | Province of Ontario | https://www.ontario.ca/laws/statute/s19005 | The most salient features of the proposed legislation relate to the creation of the new health agency, Ontario Health, and the broad authority of the Minister of Health and Long-Term Care (the “Minister”) to “integrate” health service providers. |
| 34 | Connecting Care Act | Province of Ontario | https://www.ontario.ca/laws/statute/19c05 | Legislation introducing the establishment of Ontario Health Teams, including definitions, regulations, funding and accountability, transfers, enforcements and penalties, community engagement, etc. |
| 35 | Canada Health Act | Minister of Justice | https://laws-lois.justice.gc.ca/eng/acts/c-6/page-1.html | In summary, the criteria and conditions that must be met for provinces and territories to receive federal contributions under the Canada Health Transfer include: Public administration - must be operated on a non-profit basis by a public authority; comprehensiveness - plans must cover all insured health services provided by hospitals, physicians or dentists in the case procedures take place in hospital settings; universality - all residents must be entitled to insured health services on uniform terms and conditions; portability - insured residents moving from one province or territory to another must continue to be covered for insured health services within certain conditions; and lastly accessibility of insured health services. |
| 36 | Directive – harm reduction update | City of Toronto (SSHA) | N/A | Document sent by interview participant. Updated overdose prevention and response strategies and additional measures in response to the opioid crisis and the impact of COVID-19 and physical distancing requirements in Toronto Shelter Standards (TSS) and the 24-Hour Respite Sites Standards (TRS). |
| 37 | Toronto Shelter Standards (version 4) | City of Toronto (SSHA) | Not available. Has been updated to version 5. | There are no specific guidelines on the operation of youth shelters as opposed to adult-based shelters, although it is known that the needs of both may be slightly different. - note: it is stated that the TSS are not exhaustive and is updated every 5 years - abstinence based programming section may need to be revised since the release of the SSHA directive issued in 2021. |
| **NEWS ARTICLES** |  |  |  |  |
| 38 | Ontario taking another step to integrate the healthcare system | Ministry of Health | https://news.ontario.ca/en/release/54585/ontario-taking-next-steps-to-integrate-health-care-system | The transfer of health system funding, and planning and coordination functions of the Local Health Integration Networks (LHINs) by Ontario Health will be postponed as the province responds to COVID-19. |
| 39 | Hospital Network sets aside $10M parcel of land for affordable housing | CBC News | https://www.cbc.ca/news/canada/toronto/uhn-health-care-and-homelessness-1.5287276#:~:text=Toronto-,Hospital%20network%20sets%20aside%20%2410M%20parcel%20of%20land%20for,of%20its%20low%2Dincome%20patients. | University Health Network (UHN) partners with City of Toronto and United Way on the Social Medicine Initiative to fund $10 million for land in response to the housing needs of people experiencing homelessness who come in routinely to the emergency department at Toronto Western and Toronto General hospitals.  Although not youth-specific and more general, this is being noted as a form of engagement by the health network with the city (which is part of emergency shelter management/funding) with the goal of helping to employ more housing first initiates to improve the health of this population. |
| 40 | Toronto General hospital removes bars aimed at keeping homeless people away | Global News (Canada); Author: Daniela Germano | https://globalnews.ca/news/4125132/toronto-general-hospital-homeless/ | UHN removed bars that were installed over a vent outside a downtown hospital emergency department to deter people experiencing homelessness from sleeping there (or so that was the impression that was made) - although they argue that it was because of safety concerns about garbage and needles found in the area. Executives have realized why this was not the best approach.  Not youth specific - but interesting to include because the actions by UHN in installing bars, are similar to what young people who I have interviewed have felt when visiting some of these hospitals (i.e., non-compassion, stigma, etc.). Outlines niches, organizations, and norms. |
| 41 | Hotels offer 'health and human dignity' along with a place to recover | University Health Network | https://www.uhn.ca/corporate/News/Pages/Hotels_offer_health_and_human_dignity_along_with_a_place_to_recover.aspx | Executives from hospitals have served as site managers at 2 COVID-19 recovery hotel sites in response to the outbreak emergencies at shelters. Several quotes from executives across sectors point to how cross-sector collaboration during this time led to important work that needed to be done. Some emphasis placed on need for housing first as a larger system concern.  Quote: "It's been a great example of how cross-sector partners can all come together and bring value to this work," says Wilfred Cheung, Acting Vice President, Health System Strategy, Integration and Planning with Ontario Health (Toronto). "The infrastructure that UHN provided has been critically important as have all the community providers stepping in and using their expertise to guide how best to provide care for this vulnerable population in order to deliver great care and the most positive client experience.  "When we think of where we want the healthcare system to go, this is the type of cross-sectoral partnership we want to support and encourage." |
| **SCHOLARLY ARTICLES** |  |  |  |  |
| 42 | Key attributes of integrated community-based youth service hubs for mental health: a scoping review | Cara Settipani, Lisa Hawke, Kristin Cleverley, Gloria Chaim, Amy Cheung, Kamna Mehra, Maureen Rice, Peter Szatmari, and Joanna Henderson | https://pubmed.ncbi.nlm.nih.gov/31367230/ | The scoping review identifies the key principles and characteristics of community-based, integrated youth service hubs. YouthCan IMPACT is a Toronto-based hub established in 2016, which provides a range of youth-friendly services in one setting through a rapid, stepped-care approach. Youth Wellness Hubs were noted as Ontario-wide hubs providing integrated care across 10 sites. |
| 43 | The Youth Wellness Quest: A Comprehensive Online mental health literacy and self-advocacy resource developed by youth and for youth | Asavari Syan Janice Lam, Lisa Hawke, Karleigh Darney, and Joanna Henderson | Syan A, Lam JY, Hawke LD, Darnay K, Henderson J. The Youth Wellness Quest: A Comprehensive Online Mental Health Literacy and Self-Advocacy Resource Developed by Youth for Youth. Healthc Q. 2022 Apr;24(SP):55-59. doi: 10.12927/hcq.2022.26773. PMID: 35467512. | Youth Wellness Quest resource developed by National Youth Action Council at CAMH in Toronto. The health literacy resource informs youth of possible available services, increasing their capacity to make informed mental health decisions. These are part of the Youth Wellness Hubs. |
| 44 | The Longitudinal Youth in Transition Study (LYiTS) Cohort Profile: Exploration by Hospital versus community-based mental health services | Kristin Cleverley, Julia Davies, Sarah Brennenstuhl, Kathryn Bennett, Amy Cheung, Joanna Henderson, Daphne Korczak, Paul Kurdyak, Andrea Levinson, Antonio Pignatiello, Katye Stevens, Aristotle Voineskos, and Peter Szatmari | Cleverley K, Davies J, Brennenstuhl S, Bennett KJ, Cheung A, Henderson J, Korczak DJ, Kurdyak P, Levinson A, Pignatiello A, Stevens K, Voineskos AN, Szatmari P. The Longitudinal Youth in Transition Study (LYiTS) Cohort Profile: Exploration by Hospital- Versus Community-Based Mental Health Services. Can J Psychiatry. 2022 Dec;67(12):928-938. doi: 10.1177/07067437221115947. Epub 2022 Aug 4. PMID: 35924416; PMCID: PMC9659798. | LYiTS prospectively follows youth in Toronto as they transition from child and adolescent mental health services to adult mental health services. Also examines health service utilization between youth receiving services at hospital vs. community-based mental health services. CAMH, and the hospital for Sick Kids were selected hospitals for recruitment, and the George Hull Centre and SickKids Centre for Community Mental Health were selected as community health sites. |
| 45 | “I feel like I’m a revolving door, and COVID has made it spin a lot faster”: The impact of the COVID-19 pandemic on youth experiencing homelessness in Toronto, Canada. | Amanda Noble, Benjamin Owens, Naomi Thulien, & Amanda Suleiman | Noble A, Owens B, Thulien N, Suleiman A. "I feel like I'm in a revolving door, and COVID has made it spin a lot faster": The impact of the COVID-19 pandemic on youth experiencing homelessness in Toronto, Canada. PLoS One. 2022 Aug 22;17(8):e0273502. doi: 10.1371/journal.pone.0273502. PMID: 35994505; PMCID: PMC9394800. | YEH who resided at EYSs and who moved to hotels for shelter services, and staff who worked at these sites were interviewed in the study. Some relevant organizations, and actors and their roles noted in this study, including Toronto Public Health, City of Toronto (SSHA), hotel sites, etc. |
| **OTHER DOCUMENTS** |  |  |  |  |
| 46 | Ontario’s Housing and Homelessness System | A Way Home | https://www.homelesshub.ca/sites/default/files/AWH%20Community%20Planning%20Toolkit-Appendix%20A_0.pdf | Appendix A of youth homelessness community planning toolkit |
| 47 | Housing TO: 2020- 2030 Action Plan | City of Toronto | https://www.toronto.ca/community-people/community-partners/housing-partners/housingto-2020-2030-action-plan/ | Provides a blueprint for action across the full housing spectrum - from homelessness to rental and ownership housing to long-term care for seniors. 13 key strategic actions including preventing homelessness and improving pathways to housing stability and enhancing partnerships and intergovernmental strategy are detailed in action plan through consultation with key stakeholders including groups with lived experience. Includes plan for EYSs. |
| 48 | Ontario Health's Operating Model: Patient perspective and Integrated top-line organizational structure | Matthew Anderson, President and CEO of Ontario Health | https://www.ontariohealth.ca/sites/ontariohealth/files/2020-09/OH_OpModel_and_OrgStructure_Internal_Sep92020.pdf | Summarizes July 2020 Mandate letter from Minister of Health, with the primary goal of the single agency connecting and coordinating Ontario's health care system in ways that have not been done before; and includes a framework and description of its service structure including the various portfolios involved. ** Refer to framework for understanding of new model - social service agencies, community-based care, primary care, etc. are all included in OHTs |
| 49 | Emergency Housing Action - City Council Decision | City Council | https://secure.toronto.ca/council/agenda-item.do?item=2020.PH19.11 | Outlines intergovernmental and financial approval considerations; as well as considerations to expedite the implementation of affordable supportive housing adopted by City Council.  Included because of actions including cross-sectoral organizations in EYS system and health system to help people experiencing homelessness access and maintain housing and supports. |
| 50 | Guidance Document for Harm Reduction in Shelters: A 10-point Plan | The Works, Toronto Public Health (City of Toronto) | https://www.toronto.ca/wp-content/uploads/2021/06/9633-10PointShelterHarmReduction210528AODA.pdf | Aligns with Toronto Shelter Standards, 24-hour respite standards, and 2021 harm reduction directive through City of Toronto |

## Table 2: Documents excluded from study

|  | **Document type** | **Document name** | **Organization** | **Reason for exclusion** |
| --- | --- | --- | --- | --- |
| 1 | Policy document | People’s Healthcare Act | Province of Ontario | Much is duplicate information as provided in Connecting Care Act, 2019 |
| 2 | Web page | Policies and procedures | Youth Without Shelter | Doesn’t add value to system attributes being analyzed. Only necessary if looking at systems regulations. |
| 3 | Web page | Ontario Health Agency | Ontario Health | Duplicated information to other more reputable documents. |
| 4 | Report | 500 in five: Strategic Plan 2019 -2024 | Youth Without Shelter | Does not include anything about enhancing partnerships with health sector to improve quality of care. Emphasis on education services, and youth refugee support. |
