# Supplementary material for: Bounding systems: A qualitative study exploring healthcare coordination between the emergency youth shelter system and health system in Toronto, Canada
Source: PLoS One. 2024 Jun 21;19(6):e0303655. doi: 10.1371/journal.pone.0303655 (PMC11192382; doi:10.1371/journal.pone.0303655)
Supplement: S2 File — (PDF) [file pone.0303655.s002.pdf]

Université d'Ottawa
Bureau d'éthique et d'intégrité de la recherche

University of Ottawa
Office of Research Ethics and Integrity

CERTIFICAT D'APPROBATION ÉTHIQUE | CERTIFICATE OF ETHICS APPROVAL

**Numéro du dossier / Ethics File Number**	H-12-20-5771
**Titre du projet / Project Title**	How do emergency youth shelters and the Canadian health system engage? A case study exploring coordination of care for youth experiencing homelessness in Toronto
**Type de projet / Project Type**	Thèse de doctorat / Doctoral thesis
**Statut du projet / Project Status**	Approuvé / Approved
**Date d'approbation (jj/mm/aaaa) / Approval Date (dd/mm/yyyy)**	16/04/2021
**Date d'expiration (jj/mm/aaaa) / Expiry Date (dd/mm/yyyy)**	15/04/2022

Équipe de recherche / Research Team

Chercheur / Researcher	Affiliation	Role
Alzahra HUDANI	École interdisciplinaire des sciences de la santé / Interdisciplinary School of Health Sciences	Chercheur Principal / Principal Investigator
Sanni YAYA	École de développement international et mondialisation / School of International Development and Global Studies	Superviseur / Supervisor

Conditions spéciales ou commentaires / Special conditions or comments

550, rue Cumberland, pièce 154
Ottawa (Ontario) K1N 6N5 Canada

550 Cumberland Street, Room 154
Ottawa, Ontario K1N 6N5 Canada

613-562-5387 • 613-562-5338 • ethique@uOttawa.ca / ethics@uOttawa.ca
www.recherche.uottawa.ca/deontologie | www.recherche.uottawa.ca/ethics

Université d'Ottawa
Bureau d’éthique et d’intégrité de la recherche

University of Ottawa
Office of Research Ethics and Integrity

Le Comité d’éthique de la recherche (CÉR) de l’Université d’Ottawa, opérant conformément à l’*Énoncé de politique des Trois conseils* (2014) et toutes autres lois et tous règlements applicables, a examiné et approuvé la demande d’éthique du projet de recherche ci-nommé.

L’approbation est valide pour la durée indiquée plus haut et est sujette aux conditions énumérées dans la section intitulée “Conditions Spéciales ou Commentaires”. Le formulaire « Renouvellement ou Fermeture de Projet » doit être complété quatre semaines avant la date d’échéance indiquée ci-haut afin de demander un renouvellement de cette approbation éthique ou afin de fermer le dossier.

Toutes modifications apportées au projet doivent être approuvées par le CÉR avant leur mise en place, sauf si le participant doit être retiré en raison d’un danger immédiat ou s’il s’agit d’un changement ayant trait à des éléments administratifs ou logistiques du projet. Les chercheurs doivent aviser le CÉR dans les plus brefs délais de tout changement pouvant augmenter le niveau de risque aux participants ou pouvant affecter considérablement le déroulement du projet, rapporter tout évènement imprévu ou indésirable et soumettre toute nouvelle information pouvant nuire à la conduite du projet ou à la sécurité des participants.

The University of Ottawa Research Ethics Board, which operates in accordance with the *Tri-Council Policy Statement* (2014) and other applicable laws and regulations, has examined and approved the ethics application for the above-named research project.

Ethics approval is valid for the period indicated above and is subject to the conditions listed in the section entitled “Special Conditions or Comments”. The “Renewal/Project Closure” form must be completedfour weeks before the above-referenced expiry date to request a renewal of this ethics approval or closure of the file.

Any changes made to the project must be approved by the REB before being implemented, except when necessary to remove participants from immediate endangerment or when the modification(s) only pertain to administrative or logistical components of the project. Investigators must also promptly alert the REB of any changes that increase the risk to participant(s), any changes that considerably affect the conduct of the project, all unanticipated and harmful events that occur, and new information that may negatively affect the conduct of the project or the safety of the participant(s).

Kim THOMPSON
Responsable d'éthique en recherche / Protocol Officer
Pour/For **Daniel LAGAREC** Président(e) du/ Chair of the **Comité d'éthique de la recherche en sciences de la santé et sciences / Health Sciences and Sciences Research Ethics Board**

550, rue Cumberland, pièce 154
Ottawa (Ontario) K1N 6N5 Canada

550 Cumberland Street, Room 154
Ottawa, Ontario K1N 6N5 Canada

613-562-5387 • 613-562-5338 • ethique@uOttawa.ca / ethics@uOttawa.ca
www.recherche.uottawa.ca/deontologie | www.recherche.uottawa.ca/ethics
